# Familial thrombocytopenia due to a complex structural variant resulting in a *WAC-ANKRD26* fusion transcript

**DOI:** 10.1084/jem.20210444

**Published:** 2021-04-15

**Authors:** Lara Wahlster, Jeffrey M. Verboon, Leif S. Ludwig, Susan C. Black, Wendy Luo, Kopal Garg, Richard A. Voit, Ryan L. Collins, Kiran Garimella, Maura Costello, Katherine R. Chao, Julia K. Goodrich, Stephanie P. DiTroia, Anne O’Donnell-Luria, Michael E. Talkowski, Alan D. Michelson, Alan B. Cantor, Vijay G. Sankaran

**Affiliations:** 1 Division of Hematology/Oncology, Boston Children’s Hospital, Harvard Medical School, Boston, MA; 2 Department of Pediatric Oncology, Dana-Farber Cancer Institute, Harvard Medical School, Boston, MA; 3 Broad Institute of Massachusetts Institute of Technology and Harvard, Cambridge, MA; 4 Center for Genomic Medicine, Massachusetts General Hospital, Harvard Medical School, Boston, MA

## Abstract

Advances in genome sequencing have resulted in the identification of the causes for numerous rare diseases. However, many cases remain unsolved with standard molecular analyses. We describe a family presenting with a phenotype resembling inherited thrombocytopenia 2 (THC2). THC2 is generally caused by single nucleotide variants that prevent silencing of *ANKRD26* expression during hematopoietic differentiation. Short-read whole-exome and genome sequencing approaches were unable to identify a causal variant in this family. Using long-read whole-genome sequencing, a large complex structural variant involving a paired-duplication inversion was identified. Through functional studies, we show that this structural variant results in a pathogenic gain-of-function *WAC*-*ANKRD26* fusion transcript. Our findings illustrate how complex structural variants that may be missed by conventional genome sequencing approaches can cause human disease.

## Teaser

Wahlster, Verboon, et al. identify and functionally characterize a complex structural variant in a family with thrombocytopenia that results in a *WAC*-*ANKRD26* fusion transcript encoding a shortened form of ANKRD26, whose expression during hematopoiesis is deregulated, thereby resulting in disease.

## Introduction

Recent progress in genome sequencing has enabled insights into the cause of a range of rare diseases, including numerous congenital blood disorders (Hartley et al., 2020; Liggett and Sankaran, 2020; Thaventhiran et al., 2020; Turro et al., 2020). Despite these important technological and computational advances, a substantial subset of cases remains unsolved. Complex structural variants (SVs) that alter the arrangement of large segments of the genome can be challenging to identify, especially with the use of conventional short-read sequencing approaches that are commonly employed for whole-exome or genome sequencing (Eichler, 2019). Moreover, the functional impact of specific germline SVs often remains poorly understood, particularly when these variants result in fusion transcripts or cause complex alterations beyond unambiguous loss-of-function alleles in specific genes (Eichler, 2019; Spielmann et al., 2018).

Here, we illustrate the specific challenges that can arise in identifying and characterizing a complex SV. We study a multigenerational family impacted by congenital thrombocytopenia, resembling inherited thrombocytopenia 2 (THC2; Online Mendelian Inheritance in Man accession no. 188000; Noris et al., 2011; Pippucci et al., 2011; Tan et al., 2020; Turro et al., 2020), which is typically caused by single nucleotide variants (SNVs) that de-repress *ANKRD26* expression during megakaryocytic differentiation (Bluteau et al., 2014; Marconi et al., 2017). THC2 is generally characterized by thrombocytopenia accompanied by altered hematopoiesis and predisposition to myeloid malignancies. Using long-read sequencing approaches, we assemble and fully resolve a complex SV affecting the gene implicated in THC2, *ANKRD26*. In contrast to previously described THC2 cases, this SV produces a unique fusion transcript between the first exon of *WAC* and exons 10–34 of *ANKRD26*. We demonstrate how this gene fusion acts through a gain-of-function mechanism to cause disease. More broadly, we provide an example of how commonly employed clinical variant detection and sequencing approaches can be blind to the detection of cryptic SVs that can cause human diseases.

## Results and discussion

**Figure 1.**
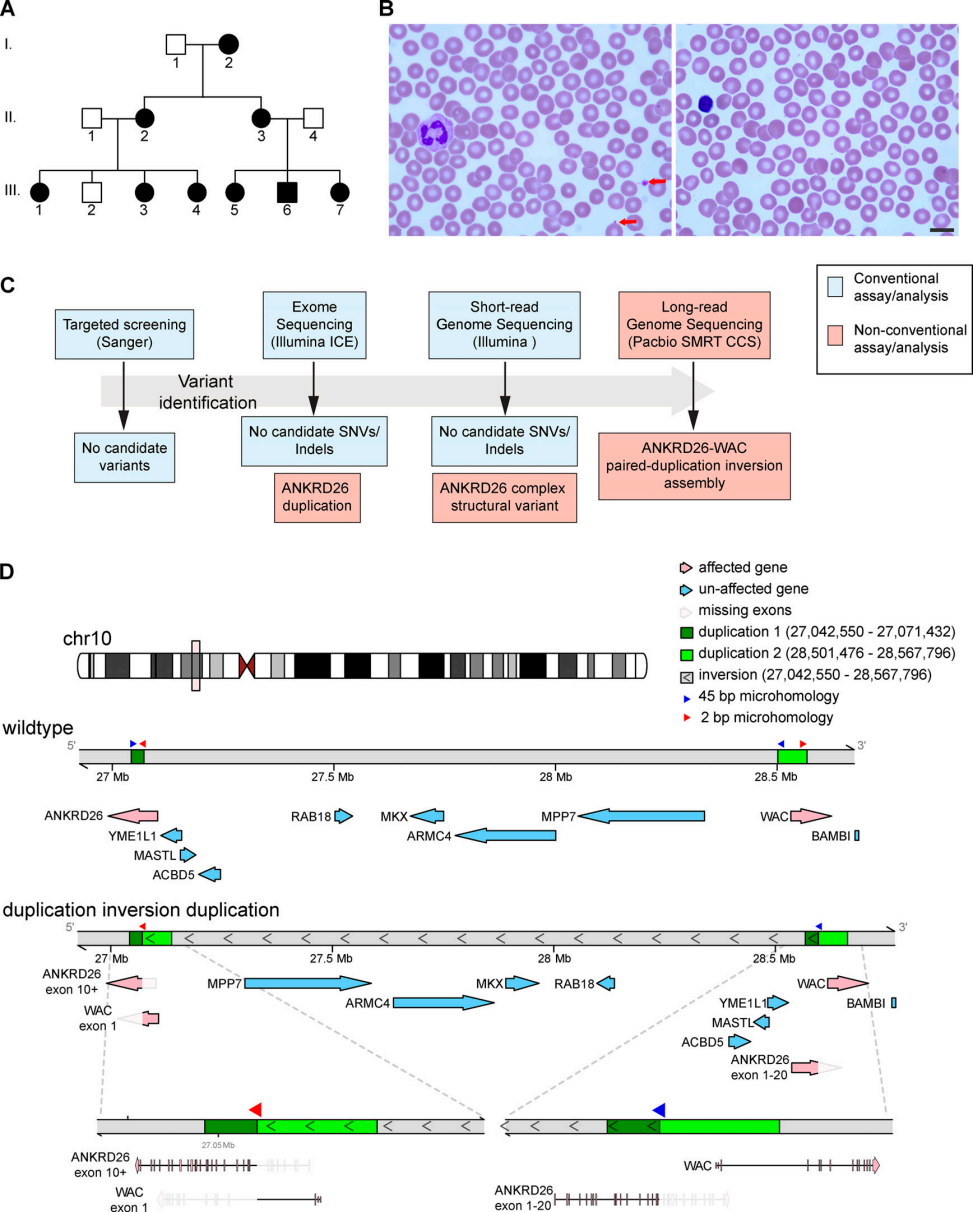
**A large paired-duplication inversion SV identified as the cause of inherited thrombocytopenia.** **(A)** Pedigree of a three-generation family affected with inherited thrombocytopenia. **(B)** Representative image of a peripheral blood smear from individual III-1, demonstrating severe thrombocytopenia with few platelets exhibiting normal granularity (red arrows). Scale bar: 10 µm. **(C)** Schematic of the genetic workup performed to identify the causal variant in this family, highlighting conventional clinical analyses (blue) versus additional approaches (red) needed to resolve this case. **(D)** The wild-type locus versus a local assembly of the paired-duplication inversion highlighting duplicated regions (greens), inverted segments (angular brackets), and microhomology regions (red and blue arrow heads) and with genes along each assembly (pink arrows denoting genes that are affected by the SV). ICE, Inosine Chemical Erasing Sequencing.

In the course of our studies of genetic blood disorders, we encountered a three-generation family where multiple members were impacted by thrombocytopenia (Fig. 1 A and Table 1). Individual I-2 had first come to clinical attention when she was 8 yr old and had developed petechiae. A low platelet count was noted at that time. At the age of 21 yr, given ongoing thrombocytopenia, a bone marrow biopsy and aspirate were undertaken, and a presumptive diagnosis of immune thrombocytopenia was made, given the presence of abundant megakaryocytes. Platelets were provided before surgeries in this individual; otherwise, no severe bleeding was noted. Two daughters of this individual, II-2 and II-3, were also found to be thrombocytopenic with easy bruising, epistaxis, and menorrhagia. However, an underlying etiology remained unidentified. Individual III-1 (the daughter of II-2) came to clinical attention for thrombocytopenia in the setting of a known family history, and a workup was initiated at our institution (Fig. 1 B). As further studies were performed, all members of this family and the children of II-3 were ascertained (Table 1). While platelet counts in this family were relatively consistent within individuals at multiple time points, the range of thrombocytopenia between family members was found to be fairly broad, consistent with previously reported cases of THC2. Individuals I-2, II-2, II-3, and III-1 presented with more severe thrombocytopenia and were more symptomatic (Table 1), whereas individuals III-5, III-6, and III-7 were found to have mild to moderate thrombocytopenia and were reported to have few bleeding-related complications. We note that there appears to be age-dependent variation in platelet count in this family, suggesting that some of the variation may be the result of age-dependent changes in hematopoiesis. Analysis of complete blood counts and peripheral blood smears of affected individuals demonstrated normal platelet size, volume, and granularity. Beyond the thrombocytopenia, no other recurrent hematologic or other medical problems were noted.

**Table 1.** Clinical characteristics of affected individuals

Individual	Age, yr^a^	Sex	Platelet count, 10^3^ cells/µl, min., max (135–400)	Platelet volume, fl (9.0–12.0)	Hematological symptoms	Hemoglobin, g/dL (11.0–15.5)	Hematocrit, % (34.0–44.5)	RBC count, 10^6^ cells/µl (3.90–5.0)	MCV, fl (82.0–98.0)	RDW, % (11.9– 14.8)	WBC count, 10^3^ cells/µl (4.0–11.0)	ANC, 10^3^ cells/µl (1.8**–**7.5)	ALC, 10^3^ cells/µl (1.0–4.8)	AMC, 10^3^ cells/µl (0.1–0.9)
I-2	75	F	22, 22.5 (0.7), 23	11.0 (0.0)	Bruising, Petechiae	12.7 (0.0)	39.6 (0.3)	4.4 (0.1)	90.8 (0.1)	14.6 (0.0)	9.8 (0.1)	5.3	3.0	0.7
II-2	45	F	23, 25.5 (2.1), 28	9.5 (1.8)	Bruising, Petechiae, Epistaxis, Menorrhagia	15.6 (0.5)	44.9 (1.8)	5.4 (0.1)	84.0 (1.3)	12.9 (0.1)	9.6 (1.6)	6.6 (2.0)	1.8 (0.3)	0.7 (0.1)
II-3	43	F	23, 25.3 (2.1), 27	10.6 (0.2)	Bruising, Petechiae, Epistaxis, Menorrhagia	13.3 (0.6)	42.7 (3.0)	4.9 (0.3)	86.4 (1.2)	12.5 (0.4)	12.5 (5.9)	10.6 (7.6)	1.8 (0.4)	0.8 (0.1)
III-1	15	F	13, 24.1 (12.2), 54	10.2 (1.4)	Bruising, Petechiae, Epistaxis, Menorrhagia	15.0 (0.8)	43.0 (2.6)	5.1 (0.4)	84.2 (2.3)	12.9 (0.6)	7.1 (1.2)	3.6 (0.7)	2.5 (0.6)	0.5 (0.1)
III-3	7	F	51, 65.5 (26.4), 105	8.7 (1.8)	Bruising, Petechiae, Epistaxis	14.5 (0.6)	37.5 (0.7)	5.0 (0.1)	74.5 (0.8)	13.0 (0.1)	17.6 (3.5)	7.0 (3.4)	8.4 (1.8)	0.8 (0.4)
III-4	5	F	70, 87.8 (33.8), 148	10.3 (0.4)	Bruising, Petechiae, Epistaxis	10.8 (0.8)	34.0 (2.5)	4.4 (0.4)	77.1 (1.0)	14.9 (0.6)	10.1 (6.6)	5.0 (2.9)	6.4 (3.6)	0.9 (0.2)
III-5	7	F	70, 85.3 (24.0), 121	8.7 (1.7)	Bruising, Epistaxis	12.1 (0.9)	35.7 (2.0)	4.7 (0.4)	76.4 (4.0)	13.3 (0.4)	13.3 (2.2)	7.3 (3.6)	3.7 (0.7)	1.2 (0.1)
III-6	5	M	72, 75 (4.3), 78	9.9 (0.4)	Bruising, Epistaxis	12.8 (0.1)	37.3 (0.4)	5.0 (0.1)	74.4 (0.1)	12.9 (0.1)	10.9 (0.2)	5.7	3.4	1.0
III-7	2	F	73, 79 (8.5), 85	10.7 (0.6)	Bruising	12.2	37.5 (0.1)	5.0 (0.1)	74.1 (0.3)	14.9	17.3 (0.2)	5.0	9.1	1.8

Data are mean (SD). Ranges in parentheses indicate the reference range of each parameter, as reported by the laboratory that performed the corresponding test. ALC, absolute lymphocyte count; AMC, absolute monocyte count; ANC, absolute neutrophil count; F, female; M, male; max, maximum; MCV, mean corpuscular volume; min., minimum; RDW, red cell distribution width; WBC, white blood cell.

a Age at time of study.

### SV identification

**Figure S1.**
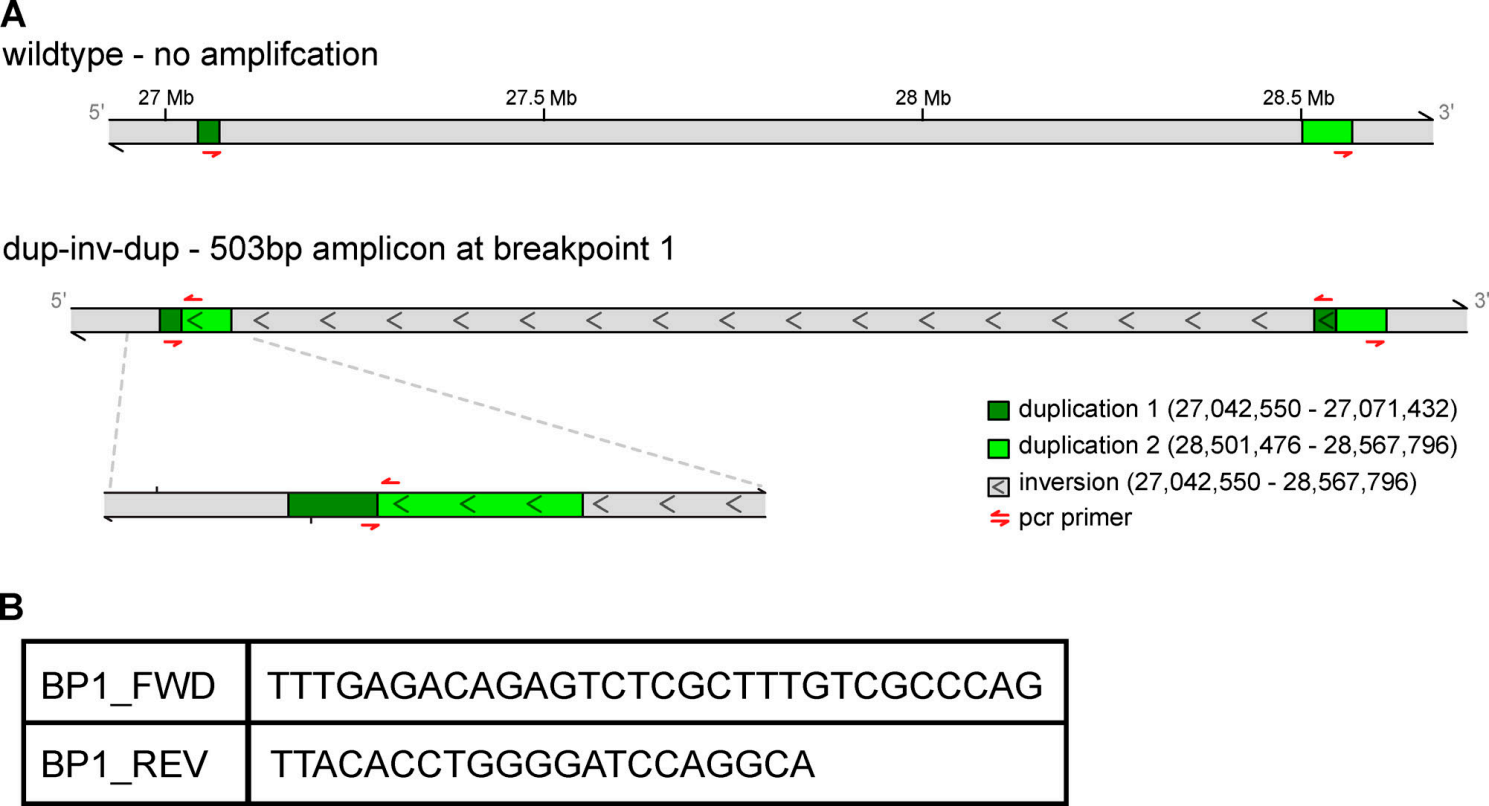
**PCR strategy to identify the causal SV.** **(A)** PCR primers were designed across breakpoint 1 such that the only possible on-target amplification occurs in affected individuals. **(B)** DNA sequence used in PCR primers is shown. dup-inv-dup, duplication-inversion-duplication.

Based on the clinical presentation of individual III-1 and other family members, targeted Sanger sequencing of *RUNX1*, *GATA1*, *MPL*, and *ANKRD26* was performed, but no pathogenic or other relevant variants were identified. Given the lack of identified mutations in this family, whole-exome sequencing of affected individuals II-2, III-1, III-3, and III-4 was performed, but no SNVs or small insertions/deletions (indels) were identified that appropriately segregated and appeared relevant to the observed blood disorder (Fig. 1 A). Additional analysis by short-read whole-genome sequencing (WGS), as is employed in standard clinical diagnostic approaches, also failed to identify other putative causal variants (Stanley et al., 2020; Ulirsch et al., 2018). Thorough reanalysis of the short-read exome sequencing data suggested increased sequencing coverage across a subset of exons in the *ANKRD26* gene, which was also noted in the short-read genome sequencing data obtained. However, these approaches did not clearly elucidate the nature of this complex SV. Therefore, by leveraging the precision of short-read genome sequencing with additional information provided by long-read genome sequencing of a trio within the larger pedigree (II-1, II-2, III-4), the duplication was determined to span exons 10–20 of *ANKRD26* and, more importantly, to be part of a larger complex SV composed of two duplications flanking a large inversion (paired-duplication inversion). These complementary technologies allowed for this SV to be precisely assembled (Fig. 1, C and D). Inspection of each breakpoint revealed microhomology between *Alu* repeats consisting of 45 and 2 bp, which is consistent with previous findings for paired-duplication inversions (Fig. 1 D; Brand et al., 2015). Short-read WGS was performed on all remaining affected family members, and breakpoints were found to be stable across all individuals over multiple generations. A PCR-based genotyping assay to assess the presence of this SV was developed and validated in affected family members and confirmed that all clinically unaffected family members who were not sequenced (e.g., III-2) did not carry this SV (Fig. S1, A and B). We found that this SV segregated appropriately in all family members with a logarithm of the odds score of 2.71 (nine individuals showing nonrecombinant phenotypes), suggesting that the cosegregation of this SV and thrombocytopenia had a <1 in 500 probability of being due to chance.

### Characterization of molecular pathogenesis of the SV

**Figure 2.**
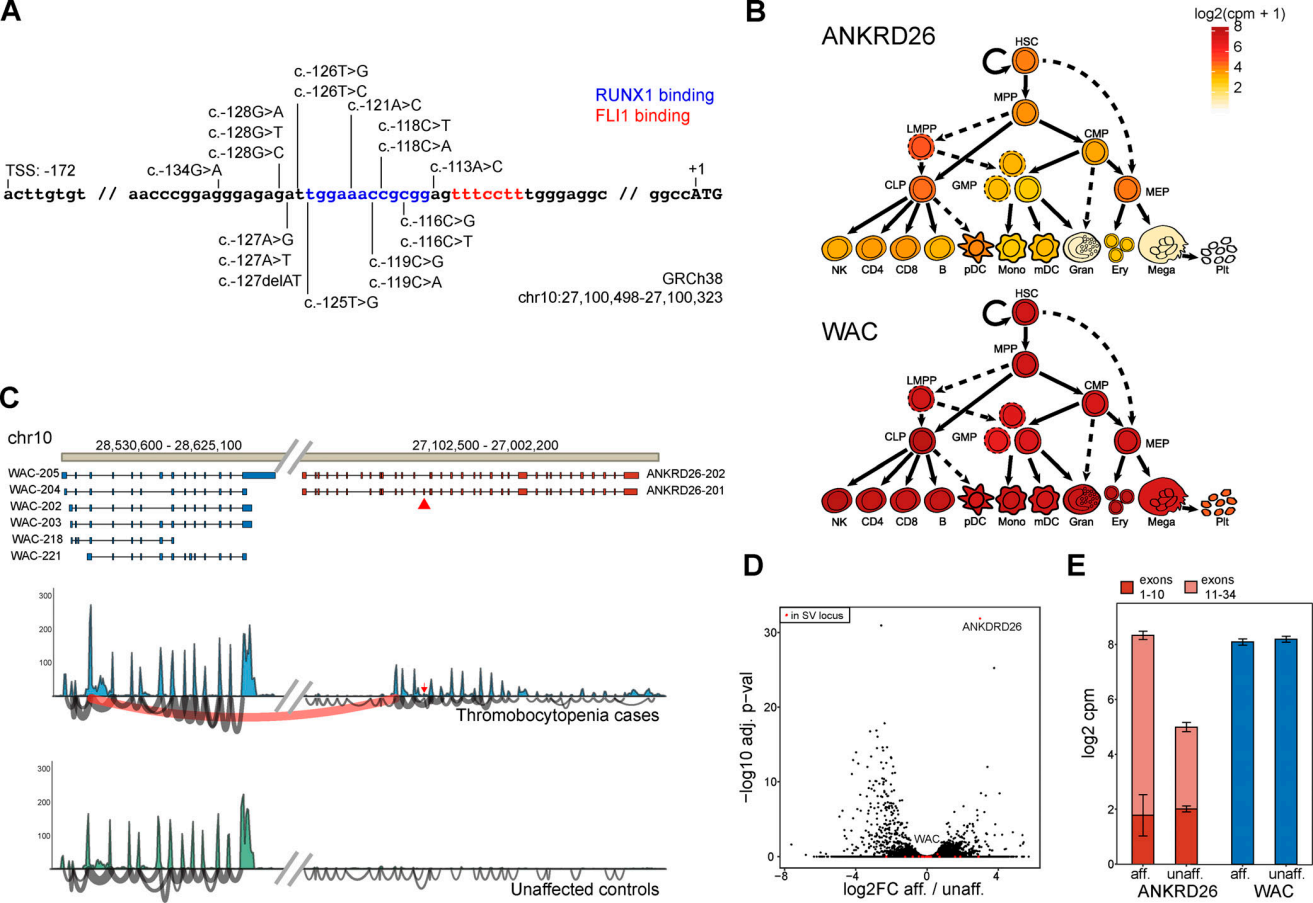
**The identified SV causes a partial transcript fusion of *WAC* and *ANKRD26* genes, leading to overexpression of a functional ANKRD26 fragment.** **(A)** Illustration of previously described *ANKRD26* promoter region variants, which lead to de-repression of *ANKRD26* and are either causal or associated with THC2. **(B)** Expression (log_2_-normalized counts per million [cpm]) of the *ANKRD26* and *WAC* genes throughout hematopoietic lineages highlights the robust ubiquitous expression of the *WAC* versus the regulated and lower expression of *ANKRD26*. **(C)** Sashimi plot of RNA sequencing transcriptomes of pooled controls versus affected individuals, highlighting the existence of a *WAC*-*ANKRD26* fusion transcript that is only present in affected individuals (top; red arc). **(D)** Volcano plot showing that *ANKRD26* is one of the most overexpressed genes in affected individuals versus controls, whereas other genes in this locus are unchanged (bottom left; red). **(E)** Bar plot of *ANKRD26* and *WAC* expression, highlighting the exons involved and not involved in the SV, shows that overexpression is due to the exons that are part of the *WAC-ANKRD26* fusion. adj. p-val, adjusted P value; aff., affected; FC, fold change; unaff., unaffected.

**Figure S2.**
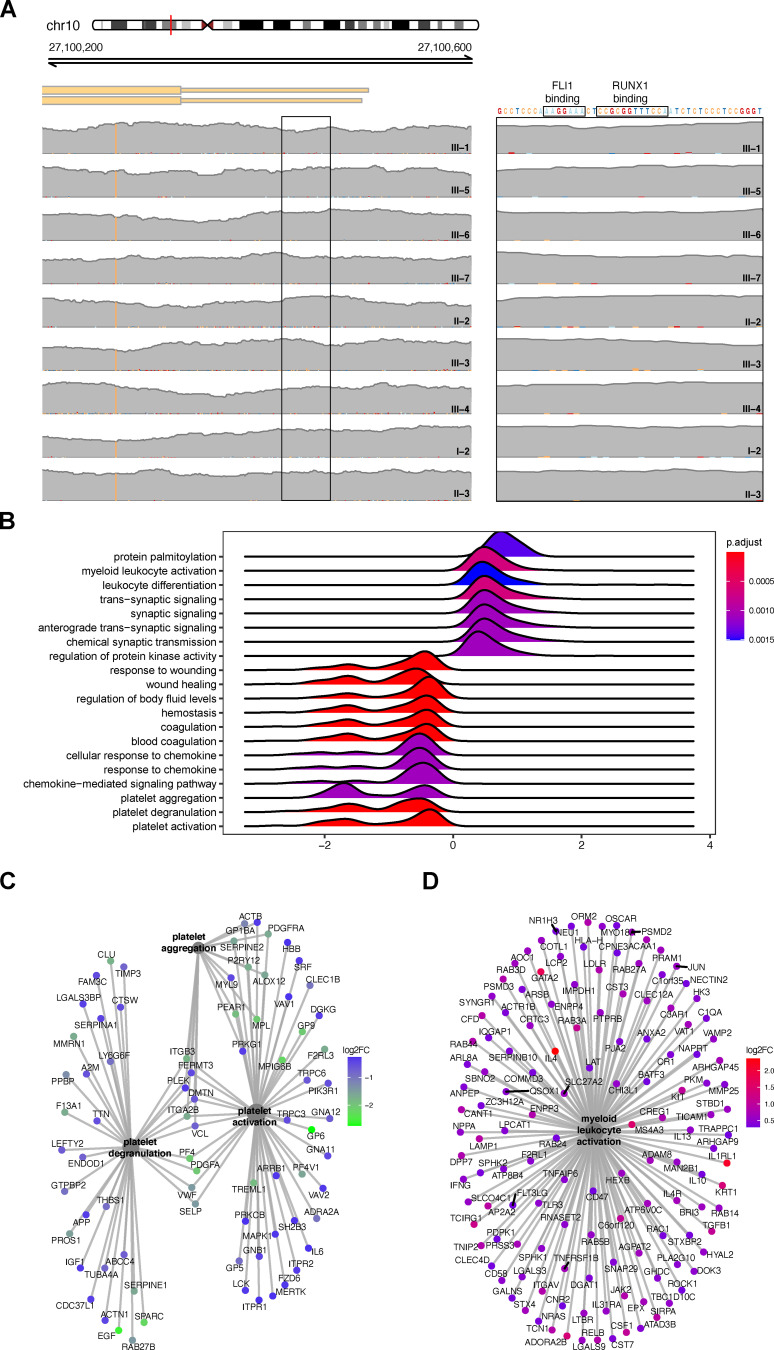
**ANKRD26 duplication-inversion-duplication, not promoter variants, cause ANKRD26 overexpression, leading to a suppression of platelet signatures and activation of signatures indicative of monocytes.** **(A)** Integrated Genomics Viewer was used to show that affected individuals harbor no variants in the *ANKRD26* promotor, which might explain the thrombocytopenia phenotype. **(B)** RidgePlot of gene expression profiles of the log_2_ fold change (FC) distributions of core genes in the top 20 most significantly enriched gene sets for affected individuals versus unaffected controls. Gene sets were limited to the biological process ontology. Distributions are colored by gene set enrichment Benjamini-Hochberg­–adjusted P values (p.adjust). **(C)** Core genes for three platelet-associated biological processes show consistent downregulation in affected individuals versus unaffected controls. **(D)** Core genes for the myeloid leukocyte biological processes show consistent upregulation in affected individuals versus unaffected controls.

To date, all pathogenic variants causing THC2 have been found in the proximal promoter of *ANKRD26*, a region that is bound by the key hematopoietic transcription factors RUNX1 and FLI1 (Fig. 2 A; Marconi et al., 2017). Binding of these transcription factors causes silencing of the *ANKRD26* gene, thereby limiting downstream thrombopoietin (TPO) signaling and enabling effective megakaryocytic maturation and proplatelet formation. The pathogenic variants identified previously prevent silencing of ANKRD26 in megakaryocytes, where it is normally repressed (Fig. 2 B; Bluteau et al., 2014). The SV present in this family, despite being large in size, only results in alterations in the structure of *ANKRD26* and the *WAC* genes, with the *WAC* gene retaining a completely intact reading frame (Fig. 1 D). Importantly, sequencing analysis confirmed the absence of pathogenic *ANKRD26* promoter variants in affected family members (Fig. 1 D and Fig. S2 A). Interestingly, we found that this SV caused the proximal promoter and exon 1 of the *WAC* gene to be juxtaposed, with exons 10–34 of *ANKRD26* resulting in an open reading frame that might enable transcription of a fusion between exon 1 of *WAC* and *ANKRD26*. Notably, the expression of *WAC* mRNA is ubiquitous within the hematopoietic system and occurs at much higher levels than *ANKRD26*, suggesting a mechanism by which overexpression of an N-terminally truncated *ANKRD26* could be achieved (Fig. 2 B). To directly assess the altered transcription occurring due to this SV, we performed RNA sequencing on peripheral blood mononuclear cells (PBMCs) from three affected probands (I-2, II-2, and II-3) and three unrelated healthy controls. Alignment of these data demonstrated large numbers of both reads where a single read spanned *WAC* exon 1 and *ANKRD26* exon 10 as well as paired reads wherein one read mapped to *WAC* and the other to *ANKRD26* (Fig. 2 C). These events were only detected in affected individuals, supporting a transcript fusion occurring due to the paired-duplication inversion (Fig. 2 C). Concomitantly, we noted upregulation of *ANKRD26* mRNA levels (∼50-fold) in the PBMCs of individuals with the SV compared with the controls, which was specific to exons that are part of the *WAC*-*ANKRD26* fusion we identified, but no significant change in expression for any other gene that was contained within or near to the SV (Fig. 2, D and E). The additional changes in gene expression likely reflect changes in the peripheral blood composition of affected individuals, who are thrombocytopenic, compared with controls, as demonstrated by reduced platelet-associated gene signatures and concomitant upregulation of myeloid cell–associated signatures (Fig. S2, C and D). Therefore, we can directly demonstrate that this pathogenic SV leads to fusion of the *WAC* promoter and exon 1 with exons 10–34 of *ANKRD26*, resulting in overexpression of the *ANKRD26* C-terminal encoding region.

### Functional analysis of fusion transcript activity

**Figure 3.**
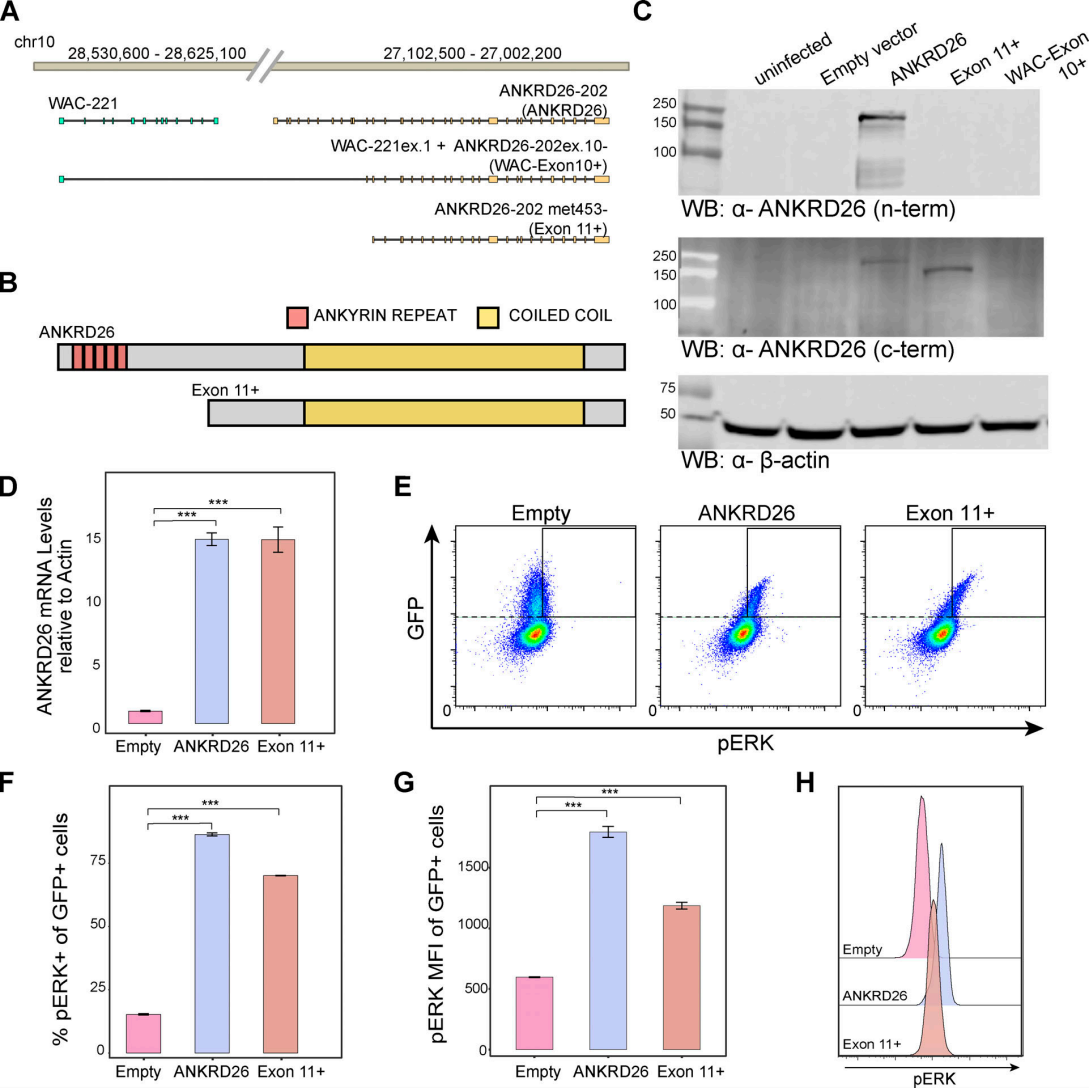
**A truncated region of ANKRD26 retains ANKRD26 functions.** **(A)** Gene models showing full-length ANKRD26, the in-frame WAC-ANKRD26 fusion, and the 5′-truncated ANKRD26 starting from Met453. **(B)** Protein models of the stably expressed full-length and N-terminally truncated ANKRD26. **(C)** Exogenous expression of full-length ANKRD26, exon 11+ cDNA, but not WAC-ANKRD26 fusion transcript can be detected, suggesting that the fusion transcript does not result in a stable protein. Protein expression levels of ANKRD26 by Western blot (WB) from whole-cell lysates of HEK 293T cells shown by probing for N- and C-terminal ANKRD26 epitopes. **(D)** RT-PCR analysis of *ANKRD26* mRNA from human HSPC sorted for GFP at 96 h after transduction with full-length *ANKRD26* and exon 11+ cDNA confirms overexpression of *ANKRD26* by 14.8 ± 0.88 and 14.79 ± 1.77, respectively. Data were normalized to the *ACTB* transcript level and represent mean ± SD of triplicates. **(E)** A representative replicate showing ERK phosphorylation assessed by flow cytometry in primary human HSPCs 96 h after lentiviral transduction with the empty control vector, full-length ANKRD26, or ANKRD26 exon 11+. **(F)** A bar plot showing quantification of three biological replicates of the percentage of pERK^+^ of GFP^+^ (successfully infected) cells (mean ± SD of triplicates shown). **(G)** A bar plot showing quantification of three biological replicates of the pERK mean fluorescence intensity (MFI) among GFP^+^ cells (mean ± SD of triplicates shown). **(H)** Representative histograms illustrating MFI of pERK within the GFP^+^ gate are shown for serum-starved HSPCs that were stimulated with TPO. A significant increase was noted for both full-length ANKRD26 and ANKRD26 exon 11+. P values were calculated by one-way ANOVA test followed by Dunnet's test (***, P < 0.001).

The resultant fusion transcripts were challenging to fully resolve, as multiple isoforms of both *WAC* and *ANKRD26* could be involved. Most potential fusion transcripts detected through RNA sequencing from *WAC* to *ANKRD26* were predicted to be out of frame for effective translation as a fusion. However, one noncanonical isoform of *WAC* (*WAC*-221) would form an in-frame fusion with exons 10–34 of *ANKRD26* (Fig. 3 A). The most likely *ANKRD26* isoform participating in this fusion is *ANKRD26*-202 based on a skipped exon with decreased coverage in the RNA sequencing data (Fig. 2 C, red arrow). Concomitantly, the fusion of all WAC transcripts with exons 10–34 of *ANKRD26* would also enable translation of a truncated form of ANKRD26 starting from a methionine located in exon 11 that would remove all ankyrin repeats but retain the coiled-coil domain (Fig. 3, A and B). Upon exogenous expression of either the full-length *ANKRD26*, exon 11 methionine initiating *ANKRD26* (herein exon 11+), or the *WAC*-*ANKRD26* fusion cDNAs in HEK 293T cells, robust protein expression could be detected from the full-length and truncated exon 11+ *ANKRD26* but not from the fusion transcript, showing that the fusion transcript was unlikely to result in a stable protein that could have pathogenic consequences (Fig. 3 C).

We then sought to assess the impact of full-length or the truncated exon 11+ *ANKRD26* in the physiologically relevant context of human hematopoiesis. We therefore introduced lentiviruses encoding these cDNAs into primary human CD34^+^ hematopoietic stem and progenitor cells (HSPCs) from healthy donors. We cultured the cells over 4 d in conditions that maintain the HSPCs. Increased *ANKRD26* expression was confirmed by RT-PCR from HSPCs sorted for GFP at 96 h after transduction (Fig. 3 D). We then starved and restimulated cells with thrombopoietin (Gibbs et al., 2011). Remarkably, we noted significantly increased levels of ERK phosphorylation with either the overexpression of full-length or the exon 11+–truncated ANKRD26 in HSPCs, whereas this was not the case with the control empty vector (Fig. 3, E–H). This finding directly demonstrates that increased and deregulated expression of a truncated form of ANKRD26 is sufficient to result in increased MAPK activation downstream of the TPO receptor and specifically in a larger fraction of HSPCs than is observed normally (Gibbs et al., 2011; Kaushansky, 2006).

### Conclusions

Here, we describe a multigenerational family affected with a phenotype resembling THC2 characterized by congenital thrombocytopenia (Pippucci et al., 2011). We identify the causal variant as a paired-duplication inversion affecting a large region involving 1.3 Mb on chromosome 10 that is significantly associated with the observed hematologic phenotype. Through molecular studies, we show that this complex SV leads to a previously undescribed *WAC-ANKRD26* fusion transcript that demonstrates gain-of-function activity in functional assays through initiation from an internal methionine in exon 11 of *ANKRD26*. Conventional WGS approaches can harbor a “blind spot” for complex SVs (Sedlazeck et al., 2018). This study illuminates the complexity in readily identifying, assembling, and defining pathogenic mechanisms for germline SVs that cause human disease. Moreover, only by RNA sequencing was it possible to prove the existence of, not just potential for, this fusion of *WAC* and *ANKRD26* that underlies the pathogenic mechanisms in the family. This emphasizes the need to consider such emerging approaches for cases unsolved using conventional WGS and annotation approaches (Eichler, 2019).

One important implication of this work is that it provides new insights into the regions of ANKRD26 that are necessary and sufficient for its pathogenic gain-of-function activity through juxtaposition with the highly active promoter of the *WAC* gene. Importantly, the truncated form of ANKRD26 that we find through this gene fusion removes the N-terminal ankyrin repeats while retaining the coiled-coil domain of this protein (Fig. 3 B). It is likely that further studies that build off these insights may lead to the identification of other pathogenic mechanisms that can impact ANKRD26 expression and thereby alter hematopoiesis to cause thrombocytopenia and increased risk for myeloid malignancies. Indeed, many current screening approaches for germline or acquired *ANKRD26* mutations in hematologic malignancies that focus on SNVs in the promoter region may miss potential pathogenic mutations (Kuo et al., 2017; Turro et al., 2020).

We also emphasize the key role that functional characterization has in delineating pathogenic mechanisms resulting from these SVs. Although the pathogenic mechanisms for a variety of complex SVs may result from loss-of-function alleles, such complex SVs can alter genome topology to cause unique changes in gene expression (Spielmann et al., 2018). Here, we provide an example of a gain-of-function fusion transcript created as a result of this complex SV. We highlight the necessity of complementary functional assays to dissect pathogenic mechanisms and emphasize the challenges ahead as a larger number of such complex SVs are recognized, assembled, and found to be associated with disease. Indeed, as our appreciation for genome variation in health and disease grows, our understanding of the enumerable mechanisms that can disrupt fundamental biological processes will continue to grow. Moreover, as advances in sequencing technologies enable identification of previously unsolved disease-associated variation, we will continue to highlight blind spots inherent in genetic technologies that currently mark the standard of clinical care.

## Materials and methods

### Study approval

This study was approved by the institutional review board at Boston Children’s Hospital, and informed consent was obtained for all individuals described. Clinical testing for pathogenic variants in *RUNX1*, *MPL*, *GATA1*, and *ANKRD26* was performed.

### Whole-exome sequencing

Whole-exome sequencing was performed with an Illumina Nextera exome capture (∼38-Mb target) and sequenced (150-bp paired reads) to cover >80% of targets at 20× and with a mean target coverage of >100×. Exome sequencing data were processed through a Picard-based pipeline, and mapping was done using the Burrows–Wheeler aligner to human genome build 38 (NCBI Assembly accession no. GRCh38.p13). Variants were called using Genome Analysis Toolkit (GATK) HaplotypeCaller package version 3.5. Putative copy number variations were called using the tool gCNV, which leverages the combined exomes of the larger Broad Institute of Massachusetts Institute of Technology and Harvard Center for Mendelian Genomics cohort.

### Short-read WGS

WGS and data processing were performed by PCR-free preparation of sample DNA (350 ng input at >2 ng/μl) using Illumina HiSeq X Ten v2 chemistry (Abdulhay et al., 2019). Libraries are sequenced to a mean target coverage of >30×. WGS data were processed through a pipeline based on Picard, using base quality score recalibration and local realignment at known indels. The Burrows–Wheeler aligner was used for mapping reads to GRCh38. SNVs and indels are jointly called across all samples using the GATK HaplotypeCaller package version 4.0. Default filters were applied to SNV and indel calls using the GATK Variant Quality Score Recalibration approach. Annotation was performed using Variant Effect Predictor. SVs were evaluated with the tools Delly, Manta, and Smoove.

### Short-read sequencing variant analysis

SNVs and indels were evaluated collaboratively using the tools seqr and gemini under a dominant inheritance model for both whole-exome and whole-genome variants. Variants with extremely low allele counts (<5) in the Genome Aggregation Database cohort were prioritized based on the rarity of the phenotype as well as the clinical prescreening for common causes of inherited thrombocytopenia. SVs were prioritized by being called by all three tools (Delly, Manta, and Smoove) and in proximity to known thrombocytopenia-associated genes.

### Long-read WGS

Long-read sequencing was performed using the Pacific Biosciences (PacBio) circular consensus sequencing (CCS) protocol. Briefly, for library preparation, 5 μg of high molecular weight genomic DNA (>50% of fragments ≥40 kb) was sheared to ∼10 kb using the Megaruptor 3 (B06010003; Diagenode), followed by DNA repair and ligation of PacBio adapters using the SMRTbell Template Prep Kit v1.0 (100-991-900). Libraries were then size selected for 10 kb ± 20% using the SageELF with 0.75% agarose cassettes (Sage Science). Following quantification with the Qubit dsDNA High Sensitivity Assay Kit (Q32854; Thermo Fisher Scientific), libraries were diluted to 50 pM per single molecule, real-time (SMRT) cell, hybridized with PacBio v2 sequencing primer, and bound with SMRT sequencing polymerase using Sequel II Binding Kit 1.0 (101-731-100). CCS sequencing was performed on the Sequel II instrument using 8M SMRT Cells (101-389-001) and Sequel II Sequencing 1.0 Kit (101-717-200), with a 2-h preextension time and 30-h movie time per SMRT cell. Initial quality filtering, base calling, and adapter marking were performed automatically on board the Sequel II to generate an initial raw “subreads.bam” file.

CCS reads were generated using CCS software v.3.4.1 from PacBio (https://github.com/PacificBiosciences/ccs) with parameters “–minPasses 3–minPredictedAccuracy 0.99–maxLength 21000,” Reads were mapped to the GRCh38 human reference genome without alternate loci. CCS reads were mapped using minimap2-2.17 (r941) with parameters “-ayYL–MD–eqx -x asm20.” SV calls were generated using pbsv v.2.2.0 (https://github.com/PacificBiosciences/pbsv) on minimap2 CCS read alignments. The pbsv discover stage was run on the whole genome using tandem repeat annotations supplied via the “–tandem-repeats” argument. The pbsv call stage was run on the full genome. The complete SV was locally assembled through manual analysis of short-read sequencing data and long-read sequencing data by evaluating split, soft-clipped, and discordant reads using both Samtools and the Integrated Genomics Viewer.

### Analysis of RNA sequencing data from peripheral blood

To assess for altered transcripts produced by the SV in affected individuals or in healthy controls, RNA was obtained from isolated PBMCs and subjected to full-length RNA sequencing analysis. For this, 4–5-ml samples of peripheral blood were obtained by venipuncture, and PBMCs were isolated via density gradient centrifugation using SeptMate columns (STEMCELL Technologies, Inc.), resulting in PBMC yields ranging from 1.5 to 3 × 10^7^ cells per individual. RNA was isolated using an RNeasy Micro Kit (QIAGEN) according to the manufacturer’s instructions. An on-column DNase digestion was performed before RNA was quantified using a Qubit RNA HS Assay Kit (Invitrogen). Approximately 10 ng of RNA was used as input to a modified SMART-seq2 protocol, and after RT, 10 cycles of PCR were used to amplify the transcriptome library (Abdulhay et al., 2019; Ludwig et al., 2019; Picelli et al., 2014). Quality of whole-transcriptome libraries was validated using a High Sensitivity DNA Chip Run on a Bioanalyzer 2100 system (Agilent) followed by library preparation using the Nextera XT kit (Illumina) and custom index primers according to the manufacturer’s instructions. Final libraries were quantified using a Qubit dsDNA HS Assay Kit (Invitrogen) and a High Sensitivity DNA Chip Run on a Bioanalyzer 2100 system (Agilent). All libraries were sequenced using Nextseq High Output Cartridge Kits and a Nextseq 500 sequencer (Illumina).

For differential expression, raw fastq files were aligned to the GRCh38 human reference genome without alternate loci with genes defined by GENCODE V22 using STAR 2.7.2b. STAR was run using the settings outFilterMultimapNmax 1–outFilterMatchNmin 35–twopassMode Basic. Gene count matrices were also generated by STAR using the setting quantMode GeneCounts. Differential expression was performed using R-based DEseq2 method, and statistical significance was determined by independent hypothesis weighting (P < 0.01). Gene fusions were detected using the tool STAR fusion using the online tool cloud computing platform Terra with all default parameters. Details are available at https://github.com/STAR-Fusion/STAR-Fusion/wiki/Terra. Gene fusions were plotted using the R package Gviz. For detailed inspection of potential fusion transcripts, all cases and all controls were pooled and visualized in Integrated Genomics Viewer.

### Hematopoietic expression analyses

Hematopoietic lineage RNA sequencing raw data were obtained from the Gene Expression Omnibus (accession nos. GSE74246, GSE58202, GSE96811, and GSE107011) for all cell lineages except megakaryocytes, which were obtained prequantified from the Blueprint Epigenomics Consortium (Stunnenberg et al., 2016). Raw data were aligned and quantified in order to facilitate downstream merging. Downstream of this protocol, expected count data from RSEM was quantile normalized. Counts were summed per cell type after normalization to prevent overweighting of more deeply sequenced samples/donors. Cells were colored in accordance with the log_2_ counts per million of the *ANKRD26* and *WAC* genes.

### Cell culture and lentiviral transduction

cDNA encoding full-length or truncated exon11+ *ANKRD26* and a *WAC*-*ANKRD26* fusion starting from exon 10 of *ANKRD26* were cloned into the HMD lentiviral backbone. For production of lentiviruses, the appropriate viral packaging and genomic vectors were introduced into HEK 293T cells with calcium phosphate transfection. Viral supernatants were collected 48 h after transfection. To examine the activity of the fusion transcript-encoded cDNAs, overexpression in HSPCs was performed by transduction of relevant constructs at a multiplicity of infection of 50. HSPCs were cultured for 5 d after transduction in serum-free StemSpan II medium (STEMCELL Technologies) supplemented with CC100 cytokine cocktail (STEMCELL Technologies), 50 ng/ml TPO (PeproTech), and 35 nM UM171 (Bao et al., 2020). To assess for effects upon signaling activity downstream of the TPO receptor, 96 h after transduction cells were serum starved for 2 h followed by stimulation with TPO at 50 ng/ml for 15 min and subjected to downstream analysis.

### Flow cytometry

After 96 h in culture, HSPCs were fixed and permeabilized according to manufacturer’s instructions and analyzed with intracellular flow cytometry using a Phosphoflow kit (BD Biosciences). Cells were stained for 60 min with pERK1/2 (Thr202/Tyr204) Alexa Fluor 647–conjugated antibodies (BD Biosciences) and subjected to flow cytometry. Flow cytometry data were analyzed with FlowJo software (version 10.6.1).

### Western blotting

Protein expression for all constructs was examined after transfection into HEK 293T and HeLa cells. Cells were harvested, washed twice with ice-cold PBS, resuspended in radioimmunoassay lysis buffer (50 mM Tris-HCl at pH 7.4, 150 mM NaCl, 0.1% SDS, 1% NP-40, 0.25% sodium deoxycholate, 1 mM dithiothreitol) supplemented with 1× Complete Protease Inhibitor Cocktail (Roche) and 1× PhosSTOP (Roche). After centrifugation at 14,000 rpm for 10 min at 4°C to remove cellular debris, whole-cell lysates were denatured at 90°C for 10 min. Equal amounts of protein were separated by SDS gel electrophoresis using 4–12% polyacrylamide gels (Bio-Rad). Subsequently, proteins were transferred onto a polyvinylidene fluoride membrane. Membranes were incubated with blocking buffer (LI-COR Biosciences) and probed with rabbit polyclonal antibody to N-terminal ANKRD26 (ab183846; Abcam), rabbit polyclonal antibody to C-terminal ANKRD26 (ab86780; Abcam), mouse monoclonal antibody to phospho-p44/42 MAPK (#9106; Cell Signaling Technology), rabbit monoclonal antibody to p44/42 MAPK (#9102; Cell Signaling Technology), and mouse monoclonal antibody to actin (sc-47778; Santa Cruz Biotechnology) all at a 1:1,000 dilution overnight at 4°C. Membranes were then washed with PBS, incubated with secondary antibodies (IRDye Secondary Antibodies; LI-COR Biosciences), and analyzed using the LI-COR Odyssey imaging system.

### Data availability

Raw fastq and processed count matrices for the RNA sequencing data are available at Genome Expression Omnibus (accession no. GSE166409). The genome and exome sequencing data files and associated metadata are available under database of Genotypes and Phenotypes accession no. phs001272 at the Genomic Analysis, Visualization, and Informatics Lab-space.

### Online supplemental material

Fig. S1 shows the PCR strategy that was used to confirm the absence of the SV in individual III-2. Fig. S2 shows sequencing data illustrating the absence of *ANKRD26* promoter variants in affected individuals and gene expression profiles demonstrating that ANKRD26 overexpression leads to a suppression of platelet gene signatures and upregulation of core myeloid leukocyte genes in affected versus unaffected controls.
